# Reverse Posterior Interosseous Artery Flap for Human Bite Injury to the Hand

**DOI:** 10.1155/2024/5392926

**Published:** 2024-02-19

**Authors:** Yusuke Hattori, Yohei Kawaguchi, Yuji Joyo, Hideki Okamoto, Hideki Murakami, Yuko Waguri-Nagaya

**Affiliations:** ^1^Department of Orthopedic Surgery, Nagoya City University Graduate School of Medical Sciences, 1 Kawasumi, Mizuho-cho, Mizuho-ku, Nagoya, Aichi 467-8601, Japan; ^2^Department of Orthopedic Surgery, Nagoya City University East Medical Center, 1 Wakamizu, Chikusa-ku, Nagoya, Aichi 464-8457, Japan

## Abstract

Bite injuries frequently occur on human hands. Human bite injuries to the hand may lead to an infection because of limited soft tissue protection and wound contamination. However, no studies have reported severe bite injuries on hands treated by flaps. We report a case of an 80-year-old woman diagnosed with a major neurocognitive disorder. The patient accidentally had a self-bite injury accompanied with an open metacarpal fracture. Debridement and fixation of the first metacarpal fracture were performed. Afterward, skin necrosis occurred gradually on the dorsum of the hand. Therefore, a reverse posterior interosseous artery (PIA) flap was used, and the postoperative course was uneventful. Given the high risk of infection, human bite injuries, particularly hand bites, should be treated immediately. Delayed treatment for such injuries may lead to extensive soft tissue defects requiring reconstruction with flaps.

## 1. Introduction

Human bite injuries frequently involve the hand, with approximately 25,000 cases reported annually in the USA [1]. Human bites can be attributed to various factors, such as aggressive behavior, sexual activities, and self-harm associated with mental illness, nervous behavior, and mental disabilities [2, 3]. Human bites can be classified as clenched (also known as fight bites, resulting from the collision between a fist and another person's teeth) or occlusive (resembling animal bites), wherein the teeth are closed over the tissue [1–3]. Owing to limited soft tissue protection, human bite injuries to the hand often lead to damage to the tendons, bones, blood vessels, and joints [2].

Human bite wounds are commonly contaminated and pose a high risk of infection [4]. Therefore, aggressive interventions, such as surgical procedures and antibiotic administration, are recommended [1–4]. However, no studies have reported bite injuries to the hands treated by flaps. Herein, we present a clinical case of a bite injury resulting from self-harm, accompanied with an open metacarpal fracture, successfully treated with a reverse posterior interosseous artery (PIA) flap. In addition, we conducted a literature review to provide an overview of this topic to clinicians.

## 2. Case Presentation

An 80-year-old woman, previously diagnosed with a major neurocognitive disorder, without other medical comorbidities, and residing in a nursing facility, was referred to our hospital due to right thumb lacerations. The lacerations had occurred 2 days prior to the patient's visit to our institution and were caused by an inadvertent self-bite. The wounds were irrigated and sutured briefly by a primary physician.

**Figure 1:**
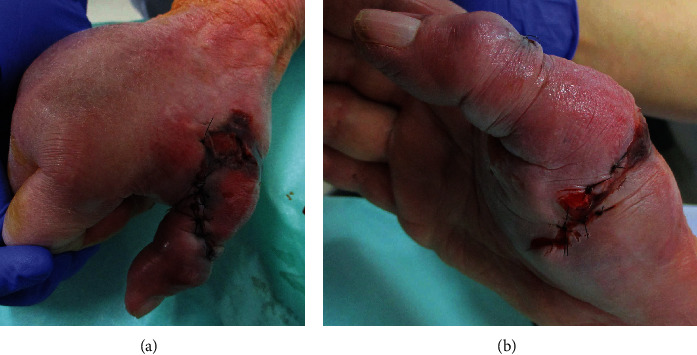
At the first visit to our hospital, open wounds were observed at the base of the right thumb. The skin around the wounds was partly necrotic.

**Figure 2:**
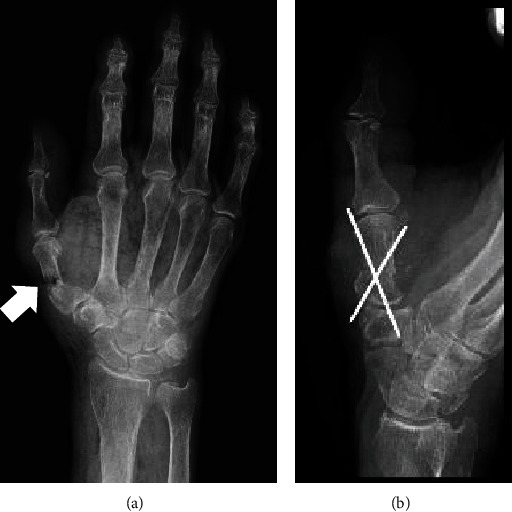
(a) The fracture in the first metacarpal shaft (arrow) is visible on the radiograph taken at the patient's first visit to our hospital. (b) The fracture was fixed by cross-pinning.

Open wounds were observed at the base of the right thumb with partial necrosis of the surrounding skin (Figure 1). Laboratory findings indicated an elevation in white blood cell count, C-reactive protein (CRP) level, and erythrocyte sedimentation rate, which revealed a left shift in neutrophils (Table 1). Plain radiography of the right hand revealed a fracture of the first metacarpal shaft (Figure 2(a)). Irrigation, debridement, and fixation of the first metacarpal fracture were immediately performed during the first visit to our hospital (Figure 2(b)). Contaminated soft tissues and bone fragments were excised, and the resected samples were cultured. *Streptococcus oralis* was detected in the culture. Therefore, ampicillin/sulbactam treatment was initiated. After the initial surgery, there was a gradual discoloration of the skin surrounding the wounds. The condition worsened, resulting in full-thickness skin necrosis. A week later, the extensor pollicis longus was exposed because of a skin defect after the second debridement.

**Figure 3:**
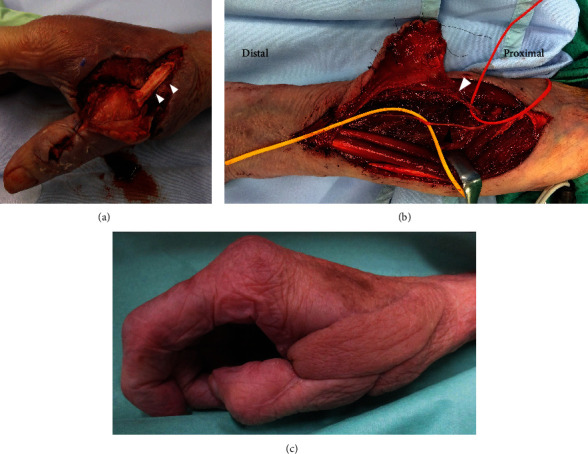
(a) A skin defect was observed in the dorsum of the hand and the first web space. Arrowheads show the extensor pollicis longus. (b) Intraoperative photograph shows the reverse posterior interosseous artery (PIA) flap before dissection of the PIA (white arrowhead). Black arrowhead shows the posterior interosseous nerve. (c) Patient outcome 1 year postoperatively.

**Figure 4:**
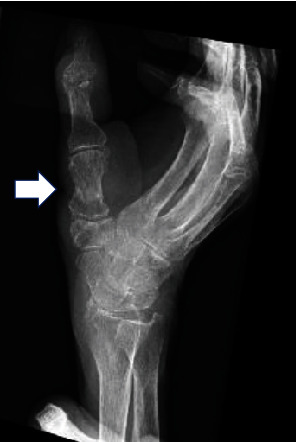
Bone union of the first metacarpal bone (arrow) is visible on a radiograph taken 1 year after the injury.

Fifteen days after the injury, we used a reverse PIA flap to provide vascularized soft tissue coverage after irrigation and debridement. The skin defect was 3.3 cm × 6 cm (Figure 3(a)). Thus, a skin island (4 × 9 cm), including three dominant perforators, was drawn over the line connecting the distal radioulnar joint (DRUJ) and the lateral humeral epicondyle. Afterward, a pivot point was marked 2 cm proximal to the DRUJ. Dissection was performed from the proximal to the distal regions. The extensor carpi ulnaris (ECU) was identified, and the fascia was incised. The fascial septum between the ECU and the extensor digiti quinti was explored to reach the vascular axis of the flap. After identifying the PIA (Figure 3(b)), it was dissected and ligated proximal to the dominant perforator. Thereafter, flap elevation was performed distally until the flap could be rotated to cover the skin defect. The donor area was closed immediately. For postoperative monitoring of the flap, an audio Doppler was used every 6 h for 3 days after surgery. No postoperative complications were observed. Bone healing was achieved 1 month after the initial surgery, and the Kirschner wires were removed. Following a 1-month administration of ampicillin/sulbactam, the patient was discharged, and minocycline (MINO) was prescribed for another month. Two months after the injury, laboratory test results were negative for CRP, and plain radiography showed bone union (Figure 4). Subsequently, the patient discontinued MINO and remained recurrence-free with a favorable prognosis (Figure 3(c)). One year postoperatively, follow-up was discontinued due to difficulties with transportation to the hospital.

## 3. Discussion

The infection rate of human bites is the second highest among all types of bites, being 15–25% [5]. Hands (25–50%) and faces (35%) are the most common anatomical sites affected by human bites [6]. Hand bites carry a higher risk of infection than face bites because of the smaller compartments of the hand and better vascularization of the face [6, 7].

The human saliva contains more than 600 bacterial species [8]. Cultures from bite wounds are usually polymicrobial and include aerobic and anaerobic isolates [1–3]. *Streptococci* (50%), *Staphylococcus aureus* (40%), and *Eikenella corrodens* (30%) are often isolated [5]. Additionally, human bites can lead to infection by blood-borne viruses, such as hepatitis B, hepatitis C, and human immunodeficiency virus [1–3].

Treatments for bite wounds of the hand depend on the wound size and infection severity. When bite wounds are complicated by bone fractures, or tendon, nerve, or vascular injuries, definitive treatment, such as internal fixation, should be performed after irrigation and debridement [6]. For soft tissue defects, various facial flaps can be used for reconstruction [9, 10]. Broad-spectrum antibiotics, such as a combination of amoxicillin and clavulanic acid, are recommended for managing human bite wounds [1, 11].

The reverse PIA flap, which is thin and pliable, provides a reliable blood supply, does not sacrifice the major forearm artery, and is suitable for reconstruction of the dorsum of the hand, wrist, and first web space [12, 13]. Our patient presented with skin defects on the dorsum of the hand and first web space. Although other flap options such as the reverse radial artery and free flaps are considered to have certain disadvantages, a reverse radial artery flap would require sacrificing the radial artery, whereas free flaps would require invasive procedures at the donor site under general anesthesia. Given the patient's old age, the reverse PIA flap was a minimally invasive flap considered appropriate for the patient. In our case, the audio Doppler was utilized for postoperative monitoring of the flap. The Doppler is a quick and useful approach for identifying and assessing the properties of the hand perforator [14]. Furthermore, osteomyelitis of the patient's first metacarpal required effective treatment. The use of the vascularized reverse PIA flap for wound coverage resulted in good outcomes at the infection site in this case.

Patients presenting 8–12 hours after bite injuries have a significantly higher risk of infection, resulting in poor treatment outcomes [3]. In our case, the wound infection during the first visit developed at least 24 h after the bite injury. Consequently, debridement and fixation of any metacarpal fractures are necessary. In the present case, a reverse PIA flap was created because of skin necrosis. Furthermore, prolonged antibiotic administration is required to treat metacarpal osteomyelitis. Human bite injuries, particularly hand bites, should be treated immediately and appropriately due to the high risk of infection.

This study had some limitations. First, the study had a small sample size. Second, the utility of the reverse PIA flap for hand reconstruction following human bite injuries remains underexplored owing to the rarity of such cases involving flap usage after bite injury. Therefore, further case studies are required to verify our findings. Despite these limitations, this study reports excellent care for severe human bite injury to the hand.

In conclusion, due to the high risk of infection, human bite injuries, particularly hand bites, should be treated appropriately and immediately. Delayed treatment of the injuries may result in extensive soft tissue defects requiring flaps.

## Figures and Tables

**Table 1:** Laboratory test findings at the patient's first visit to our hospital.

Peripheral blood		Serological tests	
Red blood cells	3.13 × 10^6^/*μ*l	CRP	10.39 mg/dl
Hemoglobin	9.9 g/dl	ESR	41 mm/1 h
Hematocrit	29.5%	HBsAg	Negative
Platelet count	282 × 10^3^/*μ*l	HCVAb	Negative
White blood cell count	12.3 × 10^3^/*μ*l	HIV	Negative
Neutrophils	91.0%		
Monocytes	4.0%		
Lymphocytes	5.0%		

CRP: C-reactive protein; ESR: erythrocyte sedimentation rate; HBsAg: hepatitis B surface antigen; HCVAb: hepatitis C virus; HIV: human immunodeficiency virus.

## Data Availability

Data sharing is not applicable to this article, as no datasets were generated or analyzed during the study.
